# Practical Notes on Diagnosis and Treatment

**Published:** 1907-09-14

**Authors:** 


### PRACTICAL NOTES ON DIAGNOSIS AND TREATMENT.

**Strychnine in Tic Douloureux.**

The patient should be kept in bed on a milk diet, and given a daily injection of strychnine, beginning with one-thirtieth and gradually increasing to one-fifth or even one-third of a grain. The treatment needs to be continued for several weeks, and for a time it may appear to aggravate the symptoms. —*Dr. D. L. Dana.*

**Paraplegia in Syphilis.**

It is a mistake to suppose that organic disease of the central nervous system only occurs as a late result of syphilitic infection. Signs of paraplegia may appear within a few months of the primary disease, and unless prompt treatment is applied may go on to permanent paralysis.

**Uranium Nitrate in Glycosuria.**

Beginning with doses of three grains freely diluted, and gradually increasing the amount, uranium nitrate has undoubtedly produced good results in many cases of glycosuria, and even in some instances of diabetes mellitus.

**Gonorrhœal Rheumatism.**

In this condition bandaging, so as to produce hyperæmia of the affected joint, has proved satisfactory. In the case of the knee one bandage is applied from the foot to the lower border of the knee joint, and a second is applied above the joint. They must be applied tightly so as temporarily to obstruct the circulation, and may be kept on from 15 minutes to one hour.

